# Compositional Dynamics of the Milk Fat Globule and Its Role in Infant Development

**DOI:** 10.3389/fped.2018.00313

**Published:** 2018-10-24

**Authors:** Hanna Lee, Emily Padhi, Yu Hasegawa, Jules Larke, Mariana Parenti, Aidong Wang, Olle Hernell, Bo Lönnerdal, Carolyn Slupsky

**Affiliations:** ^1^Department of Food Science and Technology, University of California, Davis, Davis, CA, United States; ^2^Department of Nutrition, University of California, Davis, Davis, CA, United States; ^3^Department of Clinical Sciences, Pediatrics, Umeå University, Umeå, Sweden

**Keywords:** milk fat globule, milk fat globule membrane, infant development, gut maturation, microbiota, immune system

## Abstract

Human milk is uniquely optimized for the needs of the developing infant. Its composition is complex and dynamic, driven primarily by maternal genetics, and to a lesser extent by diet and environment. One important component that is gaining attention is the milk fat globule (MFG). The MFG is composed of a triglyceride-rich core surrounded by a tri-layer membrane, also known as the milk fat globule membrane (MFGM) that originates from mammary gland epithelia. The MFGM is enriched with glycerophospholipids, sphingolipids, cholesterol, and proteins, some of which are glycosylated, and are known to exert numerous biological roles. Mounting evidence suggests that the structure of the MFG and bioactive components of the MFGM may benefit the infant by aiding in the structural and functional maturation of the gut through the provision of essential nutrients and/or regulating various cellular events during infant growth and immune education. Further, antimicrobial peptides and surface carbohydrate moieties surrounding the MFG might have a pivotal role in shaping gut microbial populations, which in turn may promote protection against immune and inflammatory diseases early in life. This review seeks to: (1) understand the components of the MFG, as well as maternal factors including genetic and lifestyle factors that influence its characteristics; (2) examine the potential role of this milk component on the intestinal immune system; and (3) delineate the mechanistic roles of the MFG in infant intestinal maturation and establishment of the microbiota in the alimentary canal.

## Introduction

Human milk has evolved to meet the unique requirements for infant growth and development and should be the sole source of nutrients for the developing infant during the first 6 months of life (1, 2). One component of milk is the milk fat globule (MFG), which is difficult to mimic in infant formulas due to its highly complex structure and variable composition. Beyond the traditional role of milk fat as a source of nutrients, which provides up to 50% of the total calories in milk, the functional importance of the MFG structure and composition on infant development is of increasing interest.

Biosynthesis of the MFG is energetically costly. Formation begins with the packaging of triacylglycerols (TAGs) into micro-lipid droplets that bud from the endoplasmic reticulum of mammary gland alveolar epithelial cells to form cytoplasmic lipid droplets (CLD) surrounded by a phospholipid monolayer. Migration of the CLD to the apical pole of the epithelial cell results in fusion with the plasma membrane and the addition of a peripheral bilayer that contains a variety of bioactive proteins and lipids (3). The fully-fledged MFG covered by the membrane (MFGM) is then secreted outside the cell to become part of the milk that provides nourishment for the infant (3, 4).

**Figure 1:**
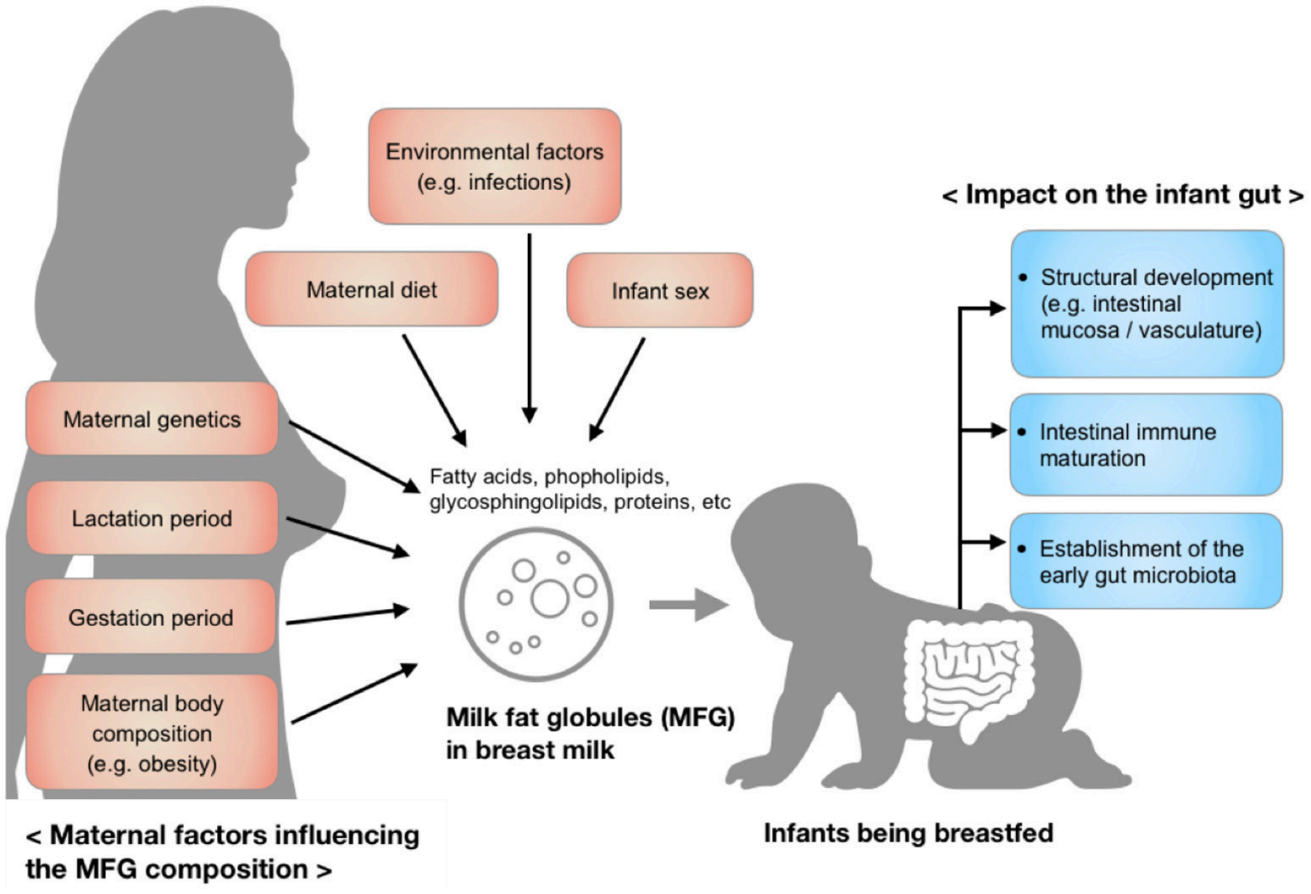
Factors influencing the composition of milk fat globules in breast milk and the impact of those components on the infant gut during early development.

MFG composition varies considerably among individuals and is dynamic over the course of lactation, but also varies over a single breastfeed (5). These variations reflect maternal factors including diet, environment, maternal genetics, and body composition, as well as the changing needs of the infant over the period of lactation (Figure 1). Recent research has shown a protective effect of MFGM against infectious diseases (6, 7), in part through the modulation of the intestinal immune response and the gut microbiota (8–10). Although the underlying mechanism is not entirely clear, the MFGM harbors two forms of glycoconjugates (glycoproteins and glycolipids), which are thought to have antimicrobial, anti-inflammatory, and prebiotic functions in the gut (11, 12). These functions may be responsible, in part, for the aforementioned modulation of immune response and microbiota.

The focus of this review is on the role maternal and environmental factors play in mediating MFG lipid and protein composition, along with inter-species differences (e.g., bovine and human), and the biological significance of the human MFG in the infant intestine, with particular attention given to structural and immune system development. The importance of this component of milk on infant health outcomes as well as identified gaps in the research literature that have been under-explored are also discussed.

## Composition of milk fat globule (MFG)

### MFG lipids

#### Structural components

MFGs are heterogeneous structures, varying in diameter, triglyceride content, and membrane and fatty acid composition (13–15). The diameter of the MFG varies between 0.2 and 15 μm, and its composition is observed to vary by size, adding more complexity to the study of their structure and function. Progress in lipids analysis has been slower than for other biomolecules, such as proteins and metabolites. This may be due partly to unsophisticated instruments, which inadequately capture the complexity of lipids, and partly to the limited amount of information that can be gleaned from genomic studies since lipid fingerprints are not directly linked to the genome (4). The core of the MFG is composed primarily of TAGs, which represent 98% of total milk fat and provides approximately half of the infant's energy intake in addition to essential fatty acids required for growth and development (16).

Milk fat contains over 400 different fatty acids, among which 15 constitute 90% of the total fatty acid pool (17). In mature human milk, the majority of TAGs in the MFG core consist of 18:1(n-9) oleic (20–35%), 16:0 palmitic (18–23%), and 18:2(n-6) linoleic (LA) (8–18%) acids (Table 1) (46). Medium chain fatty acids (MCFAs) comprise 12% of total fatty acids, and < 1% are short chain fatty acids (SCFAs) (49). Long chain polyunsaturated fatty acids (LC-PUFA), notably 20:4(n-6) arachidonic (ARA), 20:5(n-3) eicosapentaenoic (EPA), and 22:6(n-3) docosahexaenoic (DHA) acids, as well as one of the two essential fatty acids, 18:3(n-3) α-linolenic acid (ALA) are some of the least abundant, although a wide inter-individual variation exists that is dependent on maternal diet and genetics (Table 1) (50). The location of fatty acids on the glycerol backbone is highly conserved within species, but not between species, with saturated fatty acids typically occupying the *sn*-2 position of the TAG, and specifically palmitic acid occupying 50-60% of all fatty acids at the *sn*-2 position (defined as β-16:0) in human milk (51–53). The *sn*-1 and *sn*-3 positions are occupied primarily by unsaturated fatty acids, of which approximately half is oleic acid (54).

**Table 1:** Lipid composition in milk fat globules.

**Fatty acids^a^**	**% total fatty acids**	**Biological significance**
Palmitic acid (16:0)	18–23	Energy metabolism (18); used in the synthesis of other bioactive lipids (18)
Oleic acid (18:1)	20–35	Energy storage and metabolism (19); alters cell membrane fluidity (13)
Linoleic acid (n-6 18:2)	8–18	Skin barrier function (20); precursor to ARA; competes with n-3 fatty acid metabolism (21)
Linolenic acid (n-3 18:3)	0.43–1.33	Precursor to EPA and DHA (22)
Arachidonic acid (n-6 20:4)	0.36–0.49	Eicosanoid synthesis (23); neurodevelopment (24)
Eicosapentaenoic acid (n-3 20:5)	0.07–0.26	Precursor to eicosanoids (23); immune function (23)
Docosahexaenoic acid (n-3 22:6)	0.17–0.99	Cell signaling; neurodevelopment and vision (25)
**Phospholipids^b^**	**% of total phospholipids**	**Biological significance**
Phosphatidylinositol	4.6	Cell signaling; activation of Akt (26)
Phosphatidylcholine	25.2	Membrane structure; lipoprotein assembly and secretion (27)
Phosphatidylserine	5.9	Induction of apoptosis (28); carrier of DHA (29)
Phosphatidylethanolamine	28.6	Component of phospholipase D (30); cell proliferation and differentiation by regulation of pathways including MAPK and NF-kB (31)
Sphingomyelin	35.7	Metabolized to ceramide and sphingosine (32); vascular development (33); immune function (34)
**Gangliosides^c^**	14.8–26.8 mg/L in human milk	Cognitive development (35); altering membrane fluidity and function of enterocytes (36–39); cell-cell communication (40); gut maturation and immunity (41, 42)
**Cholesterol^d^**	90–150 mg/L in human milk	Structural maintenance of membranes (43); compartmentalization of membrane proteins to modulate functions (44); substrate for bile acids, vitamin D, hormones and oxysterols (45)

a Fatty acid content represents mature milk collected in nine countries (46).

b Polar lipids were quantified using HPLC-ELSD (47).

c Total ganglioside content represent Malaysian mother's milk quantified using HPLC-MS (48).

d Total cholesterol content adapted from Koletzko (45).

*ARA, Arachidonic acid; EPA, eicosapentaenoic acid; DHA, docosahexaenoic acid*.

Surrounding the TAG core is the milk fat globule membrane (MFGM), which is derived from the mammary gland epithelium (55). The MFGM is a complex mixture of 60% proteins and 40% lipids (56) and functions to stabilize the globule as an emulsion. The major building components of the MFGM are the membrane phospholipids, i.e., glycerophospholipids (glycerol-based phospholipids), which are comprised of phosphatidylethanolamine (PE), phosphatidylcholine (PC), phosphatidylserine (PS) and phosphatidylinositol (PI) (45), and sphingolipids, notably sphingomyelin (SM) (57). SM, the predominate sphingolipid in MFGM, is present in much higher quantity in human milk compared to milk from other species (e.g., bovine milk) (47). In general, PE, PC and SM are the most abundant phospholipids in the MFGM, while PS and PI are relatively minor components, although inter-individual variation does exist (47) (Table 1).

The MFGM exists as a polymorphic lipid phase with lipid-disordered domains rich in glycerophospholipids that are fluidic and lipid-ordered domains that are more rigid at body temperature (58). The lipid-ordered domains in the MFGM are called lipid rafts because cholesterol and sphingolipids interact to form circular assemblies in the outer leaflet (43). Lipid rafts, which contain approximately 80% of total milk cholesterol, play an important role in maintaining membrane structure (43), and are critical for many biological processes including compartmentalizing membrane proteins to modulate their functions (44).

Different lipid classes in the MFG exhibit distinct fatty acid profiles, with the MFGM phospholipids containing more unsaturated fatty acids than the MFG core (43). Although phospholipids in the MFGM only represent 0.5–1% of the total fat in milk, 15–20% of the total LC-PUFA (e.g., DHA and ARA) in milk is present in the MFGM phospholipids (59, 60). In contrast to MFGM phospholipids, MFGM sphingolipids are highly saturated, and are thought to maintain the lipid raft structure due to their tightly packed structure and higher melting temperature. This structure may be important in digestion, allowing for delivery of sphingosine and ceramides to the distal gut (57). The varied digestion and absorption kinetics of the MFG may be physiologically important for the infant. To date, most research has focused on understanding and modulating the overall fatty acid composition and lipid content in milk rather than studying structural function.

Glycosphingolipids (sphingolipids with a carbohydrate moiety) such as cerebrosides and gangliosides are present at low abundance (61, 62). Gangliosides are glycosphingolipids with one or more sialic acid residues, and are classified according to the number of sialic-acid residues on the molecular backbone (M = mono- or 1; D = di- or 2, as GM or GD), the number of residues attached to the sugar moiety, and the biosynthetic pathway from where they are derived (63). Human milk contains a much higher concentration of gangliosides than bovine milk (64). Supplementation of infant formula with a ganglioside-enriched dairy fraction has shown beneficial impacts on cognitive development in infants aged 0–6 months (35).

The size of the MFG is related to the TAG/phospholipid ratio, fatty acid composition (14), and cholesterol content (43). Argov et al. (65) demonstrated that smaller fat globules tend to have more phospholipids (65) that may partly result from a biosynthetic balance between phospholipids and neutral lipids coordinated in the milk-secreting epithelial cells (4). Independent of cellular TAG content, increasing intracellular phosphatidylethanolamine content has been shown to facilitate fusion between lipid droplets and hence increase the size of the MFG (66). It is also thought that the type of esterified fatty acids in the MFG core and membrane lipids contributes to globule size (67), as a higher content of LC-PUFA and medium chain fatty acids (MCFA) was observed in small fat globules in bovine milk (65, 68). Differences in digestion and fat release patterns between smaller and larger fat globules have also been described (69, 70), suggesting that varying sizes of the MFG may have distinct physiological effects. Interestingly, the mean diameter of human MFG appears to be largest in colostrum, followed by mature milk, and the smallest in transitional milk (71). Nano-sized particles termed lactosomes that are devoid of triglycerides and gangliosides but rich in phospholipids have been identified and isolated at a density equivalent to high-density lipoproteins (HDL) (higher density than native MFGs), which suggests that they may also have biological functions (65, 72).

#### Maternal factors influencing MFG lipids

- Lactation Period
  The composition of milk fat is dynamic over the course of lactation and adapts to changes in the maternal environment, diet and physiological state. While fatty acids with 4 to 14 carbons can be made from *de novo* synthesis in the mammary gland, the 16 carbon fatty acids are derived either from circulation, body stores, or diet (73). As milk matures, the average fatty acid chain length decreases because the mammary gland increases its capacity to produce MCFAs (12-14 carbons) (74). The overall LCFA content remains similar throughout lactation, with the exception of stearic acid, which is higher in colostrum (74); however, wide variations among different populations exist likely due to dietary differences (75, 76). As lactation proceeds, TAG concentrations tend to increase for the first few weeks, whereas cholesterol and cholesterol esters gradually decrease (74). Although the concentration of sphingomyelin in human milk appears to remain constant, colostrum is observed to contain more total phospholipids (75, 77) and LC-PUFAs relative to transitional and mature milk (71, 77). Yet, in most studies, including those referenced above, fatty acids in the MFG core and those in the MFGM lipids have not been separately analyzed despite the reported differences in the two fractions (59, 60).
  Levels of total gangliosides in human milk appear to be highest in colostrum (48). While the GD3 ganglioside is the predominant form in human colostrum, a shift toward GM3 predominance is observed in mature milk (64). Gangliosides contain significantly more LCFA and less MCFA in colostrum compared to mature milk (which is similar to the overall fatty acid trend in milk), as well as more monounsaturated fatty acids and less LC-PUFA (78). Distinct fatty acid esterification profiles have also been reported for human compared with bovine gangliosides (e.g., higher amounts of LCFAs longer than 20 carbons in bovine gangliosides) (79). Whether these differences translate into different health outcomes remains to be investigated.
- Genetic Factors
  Several maternal factors influence the lipid profile of human milk, and maternal genotype is a strong determinant. Within mammary epithelial cells, fatty acids activated by acyl-CoA synthase undergo a number of enzymatic reactions to produce other fatty acids, TAG, and phospholipids (4). Some of the most studied genes involved in milk lipid synthesis are those involved with the synthesis of LC-PUFA, likely due to the implication of LC-PUFA in immune responses and cognitive development in infants. These genes include fatty acid desaturase (*FADS*) genes (80–82) as well as members of the *ELOVL* family of genes that encode elongase enzymes (82, 83). However, fewer studies on maternal genetics regulating levels of phospholipid classes in the MFGM have been published. One study revealed that a polymorphism in diacylglycerol acyltransferase 1 (*DGAT1*) was associated with varying compositions of phospholipids and phospholipid/TAG ratios in bovine milk (84). Various enzymes are involved milk lipid synthesis and transport processes (4, 66, 73), which leaves considerable scope for further research on the genetic variants regulating milk lipid composition.
- Diet
  Many studies have focused on the effect of maternal diet or supplementation on milk fatty acids, and in particular LC-PUFA (16, 45, 85, 86), but how those influences are reflected in the MFG core and membrane lipids have not been clear. Milk ganglioside and phospholipid concentrations have been reported to differ by maternal geographic region within China, suggesting that diet may influence their amounts in human milk (75). Additionally, maternal socioeconomic and nutritional status have been shown to be associated with variations in total lipid and phospholipid content (87). Supplementation with LC-PUFA was also shown to increase the concentrations of total phospholipids (+18%) and sphingomyelin (+30%), as well as alter phospholipid composition of milk (59). Total choline intake, including choline supplements, have been shown to be positively correlated with breast milk phosphatidylcholine, especially at lower maternal intakes (88).
- Gestation
  Infant sex may influence milk lipid composition. Milk from mothers who gave birth to boys appeared to have higher concentrations of SM, PC, PE, and PI relative to milk from mothers who gave birth to girls (89). Associations between milk fatty acids and infection (in mother, infants, or both) were observed (90, 91), but no studies have reported such associations with milk polar lipids. Further studies into the mechanisms behind the observed relationships are warranted.

The composition of breast milk lipids appears to shift depending on whether an infant is born full-term or prematurely, including total fat (92, 93), DHA (94) and MCFA content (95). Although phospholipid composition was reported to be comparable between term and preterm milk throughout lactation (61), one study in Japan showed higher SM and lower PC in preterm mature milk relative to term mature milk (96).

### MFGM proteins

#### Structural components

The proteins of the MFG are located within the MFGM, and account for 1-4% of the total protein fraction in milk, and 1% of the total globule mass (97). Much attention has been given to the MFGM proteins, which have been extensively explored using proteomics. To date, approximately 500 proteins have been identified in human milk (98), some of which have been well characterized and include the glycosylated butyrophilin, mucins, xanthine oxidoreductase, lactadherin, CD proteins, the non-glycosylated adipophilin, and fatty acid binding proteins (Table 2). These proteins are the major proteins observed across all mammalian species, which suggests important biological functions (97, 98, 107). Interestingly, the quantities of those proteins greatly vary between species (101).

**Table 2:** Properties of major human milk fat globule membrane (MFGM) proteins.

**Protein**	**Molecular weight (kDa)**	**Location in milk fraction**	**Function**	**Difference with bovine MFGM**	**Change over lactation**	**Glycosylated (Y/N)**	**Resistance to digestion**
Butyrophilin subfamily 1 member A1 (BTN1A1)	56	MFGM	Milk fat globule secretion, immune system	Higher in human than in bovine MFGM^a^	Higher in mature MFGM than in colostrum MFGM^a^^,^^c^	Y	Rapidly digested in the infant stomach^f^ but more resistant to pepsin compared to XOR^g^; well digested by trypsin and by pronase E^h^
Mucin 1 (MUC 1/PAS 0)	250-450	MFGM	Immune protection	Lower in human than in bovine MFGM, but not significant (*P* > 0.05)^b^	No significant change reported in human MFGM; but in bovine MFGM higher at d7 (7.7-fold) compare to colostrum^d^	Y	Significantly resistant to gastric digestion and may survive to the distal gut^f^^,^^i^; detected in feces of breastfed infants
Mucin 4 (MUC 4)	232	MFGM	Immune protection	Higher in human MFGM (*P* < 0.05); not detected in bovine MFGM^b^	No significant change reported^c^	Y	Not specified, but likely be resistant to digestion due to the heavy glycosylation as glycoproteins tend to be resistant to proteases relative to non-glycoproteins^j^
Xanthine oxidase (XDH/XO, XOR)	145	MFGM	Milk fat globule secretion, immune system	Lower in human than in bovine MFGM^e^; but not significant in another study (*P* > 0.05)^b^	Highest at 6 months during 12 months lactation^c^	Y	Resistant to hydrolysis by trypsin and partially attacked by pronase E^h^
Lactadherin (PAS VI/VII, MFG-E8)	43	MFGM	Immune system	Lower in human than in bovine MFGM (*P* < 0.05)^b^	No significant change reported^c^	Y	Resistant to human neonatal gastric juice digestion at pH 4 (bovine lactadherin)^i^; detected intact in gastric aspirate samples of preterm-infants^f^; resistant to hydrolysis by trypsin and partially attacked by pronase E^h^
Cluster of differentiation 14 (CD14)	40	MFGM	Immune system	Higher in human than in bovine MFGM (*P* < 0.05)^b^ ; CD36 was dominant in bovine MFGM^d^	*Not specified*	Y	Resistant to pepsin^j^
Adipophilin (ADPH)	52	MFGM	Milk fat globule secretion	*Not specified*	No significant change reported in human MFGM; but in bovine MFGM 3.4-fold upregulated at day 7 compared to colostrum^d^	N	Well digested by trypsin and by pronase E^h^
Fatty-acid binding protein (FABP)	13	Whey and MFGM	Fatty acid transport, milk fat globule lipid synthesis,	Higher in human than in bovine MFGM *(no P value reported*)^e^	Higher at later lactation^c^	N	*Not specified*

a Yang et al. (99),

b Hettinga et al. (1),

c Liao et al. (97),

d Reinhardt and Lippolis (100),

e Yang et al. (101),

f Peterson et al. (102),

g Ye et al. (103),

h Vanderghem et al. (104),

i Chatterton et al. (105), and

j *Le et al. (106). Note that the study by ^d^Reinhardt and Lippolis (100) used bovine MFGM*.

Human MFGM proteins were first separated by 2-demensional electrophoresis in 1997 (108) and besides the common MFGM proteins (mentioned above), the following proteins have been frequently identified in human MFGM in several proteomics studies: α-lactalbumin, lysozyme, β-casein, clusterin, lactoferrin, Immunoglobulins (e.g., IgA α-chain), tenascin, apolipoproteins (e.g., type A-I) and fatty acid synthase (1, 97, 108, 109) (Table 3).

**Table 3:** Properties of minor human milk fat globule membrane (MFGM) proteins.

**Protein**	**Molecular weight (kDa)**	**Location in milk fraction**	**Function**	**Difference with bovine MFGM**	**Change over lactation**	**Glycosylated (Y/N)**	**Resistance to digestion**
Carbonic anhydrase 6	35	MFGM	Acid neutralizer, antibacterial component, and growth factor	*Not specified*	No significant change reported^c^, or 8-fold higher in colostrum MFGM than the MFGM in mature milk^e^	Y	*Not specified*
Milk alkaline phosphatase (AP)	86	MFGM	Immune system	*Not specified*	*Not specified*	Y	*Not specified*
Lysozyme	17	Predominantly in whey and to lesser extent in MFGM	Antibacterial component, immune system	Higher in human than in bovine MFGM (*P* < 0.05)^b^	Higher at later stages of lactation^a^^,^^c^	Y	Not detected in feces of breastfed infants^i^; resistant to pepsin, but susceptible to trypsin^j^
Lactoferrin	78	Predominantly in whey and to lesser extent in MFGM	Antibacterial component, immune system	Higher in human than in bovine MFGM (*P* < 0.05)^b^	No significant change reported^c^; however, higher in early milk than in mature milk^f^	Y	4-9 % of ingested lactoferrin detected in feces of breastfed infants^i^
Osteopontin (OPN)	41-75	Predominantly in whey and to lesser extent in MFGM	Antibacterial component, immune system	Lower in human than in bovine MFGM but not significant (*P* > 0.05)^b^	No significant change reported^a^^,^^c^; but in bovine milk, higher in early milk MFGM^d^	Y	Partially resistant to proteolysis when incubated with infant gastric juice *in vitro*^k^
α-Lactalbumin	16	Predominantly in whey and to lesser extent in MFGM	Antibacterial component, immune system	Higher in human than in bovine MFGM (*P* < 0.05)^a^	Higher in mature human MFGM than colostrum MFGM^a^; or no change reported^c^.	Y	Digested in the small intestine, releasing bioactive peptides and essential amino acids^l^
Immunoglobulins (e.g., IgA α-chain C region)	37-38	Predominantly in whey and to lesser extent in MFGM	Antibacterial component, immune system	A wider range of Ig present in human MFGM; IgA is more enriched in human than in bovine MFGM^b^	IgG H chain, Ig heavy chain variable region, polymeric immunoglobulin receptor and immunoglobulin J chain were higher in colostrum^a^	Y	Resistant to digestion and survived intact to the stool^m^
Toll-like receptors (e.g.,TLR2, 4)	~90	MFGM	Antibacterial component, immune system	Higher TLR2 in human than in bovine MFGM^g^	Higher TLR4 in mature milk MFGM than in colostrum MFGM^h^	Y	*Not specified*
Clusterin	52	MFGM	Antibacterial component	Higher in human than in bovine MFGM (*P* < 0.05)^b^^,^^g^; but another study reported lower quantity in human than in bovine MFGM^a^	No significant change reported^a^; but in bovine, colostrum MFGM has significantly higher quantity than mature MFGM^b^	Y	Resistant to gastric hydrolysis^n^
Tenascin	241	Whey and MFGM	Antibacterial component	*Not specified*	Significantly higher in colostrum MFGM than in mature MFGM (*P* < 0.05)^a^^,^^c^	Y	Resistant to gastric hydrolysis^n^

a Yang et al. (99),

b Hettinga et al. (1),

c Liao et al. (97),

d Reinhardt and Lippolis (100),

e Karhumaa et al. (110),

f Rai et al. (111),

g Lu et al. (107),

h Cao et al. (98),

i Davidson and Lonnerdal (112),

j Hamosh (113),

k Demmelmair et al. (114),

l Layman et al. (115),

m Demers-Mathieu et al. (116), and

n Dallas et al. (117).

*Note that the study by Reinhardt and Lippolis (100) used bovine MFGM*.

BTNs are members of the immunoglobulin (Ig) superfamily, and it is the butyrophilin subfamily 1 member A1 (BTN1A1) that has been shown to be associated with human MFGM (97). The structures of the BTNs and their functions have been reviewed elsewhere (118). Another abundant class of proteins in human MFGM is the mucins (MUC), of which Mucin 1 (MUC1) and MUC4 are the most abundant. MUC proteins have highly glycosylated extracellular domains, which makes them resistant to digestion (119), and potentially available to act as decoys for pathogens (described below).

Xanthine oxidoreductase (XOR) is another major protein (120) with a critical role in milk fat secretion. XOR aids with the fusion of the apical plasma membrane onto the fat globules through structural interactions with BTN and adipophilin (ADPH) as a tripartite structure (121). XOR is a highly conserved molybdoenzyme that oxidizes a wide range of substrates (generally with low specificity) including purine nucleotides and has been suggested as an antibacterial component in the MFGM (122). Loss of XOR has been shown to result in less efficient milk fat secretion in mice (123).

Other glycosylated MFGM proteins include milk fat globule-epidermal growth factor 8 protein (MFG-E8; PAS VI/VII), also known as lactadherin (124). Human lactadherin was first identified in the MFGM as well as in the lactating mammary gland but was recently also found in other tissues such as the endometrial epithelium (125). It has been implicated in the autoimmune disease systemic lupus erythematosus (126), and may have a role in sepsis (127).

The well-described non-glycosylated MFGM proteins include adipophilin (ADPH), and fatty acid binding proteins (FABP). ADPH (also known as perilipin 2) is in the perilipin family of proteins that regulate lipolysis by controlling the access of proteins to the lipid droplet surface (128). A recent study using homology modeling suggested that the ADPH C-terminus forms a four-helix bundle motif which aids in formation of a stable membrane bilayer during lipid secretion (shown in mice and *in vitro*) (129). FABP is involved in the intracellular transport of fatty acids, which is a critical step in the synthesis of MFG lipid constituents such as TAG and phospholipids (4).

Compared to the aforementioned major MFGM proteins, hundreds of other proteins with lower abundance exist, which include the glycosylated enzymes carbonic anhydrase, milk alkaline phosphatase (AP), lactoferrin, osteopontin (OPN) and lysozyme (109, 130, 131), while the last three are present primarily in milk whey and to a lesser extent in the MFGM. Carbonic anhydrase, also present in saliva, serum and tissues, has a few proposed functions: acid neutralizer, antibacterial agent (130) and growth factor (132), yet clinical significance as a milk component has not been established. Milk AP is an enzyme derived from the membrane of the mammary gland epithelial cell and is covalently bound to phosphatidylinositol of the MFGM (133). This same enzyme was shown to be expressed in human liver, where zinc and magnesium are required for maximal activity (134). Lactoferrin, a member of transferrin family, was first identified in milk but is also found in most exocrine fluids of mammals (e.g., saliva, tears and bile) (135). Lactoferrin is a multifunctional protein and a key component in innate immunity (136, 137). It has also been shown to improve neurodevelopment in a piglet model (131). Charlwood et al. identified α-lactalbumin and β-casein in MFGM isolates (109), although incorporation of whey and casein proteins to the MFGM via sulfhydryl–disulfide interchange reaction during the isolation could be the origin (138). OPN is present in the MFGM as a minor constituent (97). It makes a protein complex with lactoferrin *via* electrostatic interaction, potentially preventing it from being digested (139) as OPN is resistant to proteolysis by infant gastric juice *in vitro* (105). Several enzymes including a 5′-nucleotidase, an ATPase, and a nucleotide pyrophosphatase that are known to be localized in liver plasma membranes have also been identified in the MFGM (55).

#### Maternal factors influencing MFGM proteins

- Lactation Period
  As the MFGM is derived from the mammary gland epithelial cell, mammary gland cell biology, which varies over the course of lactation, is captured in the MFGM proteome. From postpartum day 1–7, significant increases in bovine MFGM proteins related to lipid synthesis (e.g., acyl-CoA synthetase, lanosterol synthase, lysophosphatidic acid acyltransferase and FABP) and secretion (e.g., the tripartite complex; BTN, APN and XOR), as well as mucins (MUC1 and 15) have been observed, suggesting a developmental shift to increase efficiency of milk lipid secretion (100). In contrast, apolipoproteins (e.g., A1, C-III, E, and A-IV) and immune-related proteins (e.g., immunoglobulin γ1 chain C region, clusterin and lactoferrin) have been observed to decrease (100) in bovine MFGM.
  Analysis of human milk revealed that as lactation proceeds (from 0 to 6 months of exclusive breastfeeding), levels of proteins related to lipid synthesis and transfer (e.g., FABP, nonspecific lipid transfer protein, and proactivator polypeptide), intracellular folate uptake (e.g., folate receptor-α), actin filament organization (e.g., gelsolin and heat shock protein beta-1), antioxidant function (e.g., glutathione peroxidase 3), and antimicrobial function (e.g., lysozyme C) were found to increase in the MFGM (97). BTN and XOR levels were also shown to increase over the first 6 months of exclusive breastfeeding but tended to decrease during partial weaning (from 6 to 12 months) (97). No significant changes have been observed for MUC4, lactadherin, carbonic anhydrase 6, and lactoferrin in human MFGM (97). However, another study showed significantly higher levels of carbonic anhydrase 6 in human colostrum compared to mature milk (110). In contrast, the levels of proteins with potential antimicrobial function, including human leukocyte antigen (HLA) II (which aids antigen presentation to T cells) (97) and AP (140), were found to be higher in colostrum compared to mature human milk, which may possibly compensate for immature neonatal immunity (discussed in section Intestinal Immune Maturation). Compared to bovine MFGM, the human MFGM proteome has been less explored (Tables 2, 3).
- Environmental Factors
  Proteins in the MFGM have been associated with various health benefits, particularly in immune defense (97). In addition to gradual changes during lactation, temporal fluctuations in the immune-related proteins of the MFGM have been observed during immune challenges such as bacterial infection. For example, the infectious bacterium *Mycoplasma agalactiae*, which causes mastitis, initiates an immune response involving up-regulation of proteins involved in host defense, inflammation, and oxidative stress, and down-regulation of proteins involved in milk fat metabolism and secretion in lactating ewes (141). Similarly, in lactating cows infected with *Staphylococcus aureus*, neutrophil extracellular traps (NETs), which are known to amplify bactericidal properties of antimicrobial peptides, were observed to accumulate in the MFGM fraction to a greater extent than observed in the whey or milk exosome proteins (142). Although these were shown in other species, similar phenomena likely occur in human MFGM reflecting the mammary gland immune response. Whether those proteins will still be active in the intestine of breast-fed infants remains to be determined, and in this sense, their metabolic fates in the GI tract are worth investigating.
- Species
  So far, only a few studies have reported (1, 98, 99, 101, 107) or summarized (143) cross-species comparison of the MFGM proteome (Tables 2, 3). One such study revealed that several MFGM proteins involved in lipid and fatty acid catabolism are higher in relative abundance or uniquely found in human milk compared with milk from other mammalian species. These proteins include peroxisomal acyl-coenzyme A oxidase 3, bile salt-stimulated lipase (BSSL), peroxisomal bifunctional enzyme, peroxisomal multifunctional enzyme type 2, hormone-sensitive lipase, lipoprotein lipase and sphingomyelin phosphodiesterase, all of which as isolates *in vitro* retain their lipolytic activity (107). BSSL has demonstrated roles in immune-modulation, intestinal growth (144), and antimicrobial action (145).

Variable expression of host defense proteins has also been observed across different species. Human MFGM is enriched with MUC4 and TLR2 relative to MFGMs from other mammals (bovine, goat and yak), which may improve innate immune response and protection against gram-positive pathogens (107). Compared to bovine MFGM, human MFGM is significantly enriched with lactoferrin, whereas cathelicidins (antimicrobial peptides) appear to be uniquely found in bovine MFGM (107). In another study, Yang et al. reported that human MFGM (pooled from 10 mothers between 3 and 8 months post-partem) was significantly more enriched with FABP but much less in XOR compared to pooled bovine MFGM (101). Importantly, bovine MFGM appears to exhibit a wider range of proteins with antibacterial properties (e.g., cathelicidins), whereas human MFGM was more enriched with the proteins involved in mucosal immunity (i.e., IgA, CD14, lactoferrin, and lysozyme) (1).

Commercially available MFGM isolates (e.g., used in infant formulas) are predominantly bovine sourced. It is therefore of great interest to understand the differences between human and bovine MFGM (and therefore the functions) to fully understand what functions are missing from bovine MFGM. Variations in isoforms and glycoforms of MFGM proteins exist within and/or between species, although less explored (146–148), which may contribute to differences in molecular functions such as binding, receptor activity, signaling, and enzyme activity. This aspect is beyond the scope of this review but further comparative studies would greatly benefit the field and promote its application.

## Function of MFG in infant gut maturation

In addition to its role in digestion and nutrient absorption, the gastrointestinal (GI) tract functions as a critical first defense immunological barrier. Gut maturation is stimulated by constant interactions between dietary components, endogenous secretions, host gastrointestinal cells, and microorganisms, all of which contribute to the development of intestinal morphology, immune function and composition of the gut microbiota. The “critical window” hypothesis postulates that events occurring early in life that disrupt the microbial ecology of the young gut, increase the risk of developing disease later in life (149). In view of this, the mechanisms through which breast milk guides early intestinal development while protecting against potentially harmful insults has been a key research question. The MFG may have a critical role in intestinal development. However, the MFG has been historically removed from breast milk substitutes (8). This section aims to review the primary roles of the MFG and its components in the development of: (1) the intestinal structure; (2) the intestinal immune system; and (3) the gut microbial community structure, with a focus on infancy.

### Structural development of the intestine

Intestinal growth and maturation begin *in utero* and continues postnatally. During this period, intestinal development is characterized by active tissue growth and morphological changes (150). Human milk lipids contain numerous components that aid in the postnatal development of the intestinal mucosa, vasculature, and motility. Studies on human infants are limited due to the invasive nature of the procedures involved, and therefore the bulk of available research utilizes animal models, and particularly the piglet model, which has physiological similarity with humans (151).

- Development of the Small Intestinal Mucosa
  Milk lipids appear to improve intestinal integrity by serving as essential building blocks for cellular membrane structure and as signaling messengers for cell growth, proliferation and migration (8). It was recently reported that the addition of bovine MFGM to a control formula in rat pups accelerated intestinal development and improved intestinal mucosal architecture by improving epithelial cell proliferation and differentiation, as well as expression of tight junction proteins to levels similar to mother-reared pups (152). These findings are consistent with another study that showed feeding an MFGM-rich post-weaning diet to mice strengthened the mucosal barrier by protecting against LPS-induced gut leakiness (153). Recently it was shown in a Caco-2 cell model that addition of polar lipids derived from bovine milk in the form of beta serum concentrate mitigated damage caused by a TNF-α challenge to the intestinal epithelial barrier (154). It is possible that MFGM aids in the maturation of the gut through both direct and indirect modulation of the gut microbiota, particularly since MFGM supplementation is observed to have the greatest effect on the colon (154), which is the site harboring the highest density of microbes.
  The MFGM is a carrier of polar lipids, whose digestive products are essential for the morphological and functional development of the newborn intestine. The MFGM is an exclusive carrier of gangliosides to the neonatal gut (42), and a ganglioside-enriched diet has been shown to significantly increase total ganglioside and GD3 content, while decreasing GM3 and reducing the ratio of cholesterol to ganglioside in the enterocyte membrane of rat pups (36). These findings suggest that gangliosides can be incorporated into the intestinal mucosa, where they can alter membrane fluidity and enterocyte function (36).
  Gangliosides are integral components in cell membranes, and the oligosaccharide residues that extend from the cell surface serve as surface markers in cell-cell communication (40). Gangliosides may also modify the brush border membrane of the GI tract. For instance, when dietary bovine gangliosides were provided to weanling rats, multiple changes in the intestinal epithelium were observed, including an increase in the content of ether phospholipids (a group of phospholipids with an alkyl or alkenyl bond at the *sn*-1 position) (39), greater incorporation of LC-PUFAs such as DHA and ARA (38), and enhanced LCFA uptake (37). In an *ex vivo* study, pre-exposure of infant bowel tissue to gangliosides reduced bowel necrosis and pro-inflammatory signals in response to LPS, implying an *in vivo* functional re-structuring of enterocytes (155).
  Sphingolipids such as SM that are present in the MFGM and in the intestinal apical membrane are digested by brush border enzymes (expressed at birth) to generate the digestive products: ceramide, sphingosine, sphingosine-1-phosphate (S1P), and ceramide-1-phosphate (8). These metabolic products are known to mediate intracellular signaling pathways that are involved in cell growth, differentiation, apoptosis and immune cell migration in the neonatal mucosa (8, 156), and to facilitate enzymatic and morphological maturation of the intestine (157). The importance of sphingolipids and gangliosides in infant gut maturation, immunity, and neurological development has been reviewed elsewhere (42).
  In newborns, PC and SM in MFGM are important sources of choline, which is an essential component of cell membranes, neurotransmitters (e.g., acetylcholine), and for neurogenesis and synaptogenesis (8). Phospholipids carry essential LC-PUFAs, critical molecules for membrane fluidity of the intestinal mucosa or neuronal tissues (57). The benefits of phospholipids in human milk have been broadly discussed (158).
- Development of Intestinal Vasculature
  During the first month of life, intestinal tissues expand and develop, and intestinal perfusion increases as a means to supply sufficient oxygen to accommodate these increases in metabolic activity. Vascular tone is mediated by endothelial nitric oxide synthase (eNOS) and the primary constrictor stimulus, endothelin-1 (ET-1), which promotes vasodilation and increases vascular resistance, respectively [reviewed in Nowicki (159)]. In addition to these endogenous regulators, SM and its metabolites (mentioned above) are able to alter infant vasculature, thereby influencing gut maturation. For example, S1P activates Akt signaling (a protein kinase with many regulatory functions in the cell), which initiates angiogenesis by invoking endothelial cell migration and morphogenesis (33). Additionally, S1P increases vascular barrier function by up-regulating adherence junctions (160, 161). Importantly, S1P levels are metabolically regulated by adiponectin, another component of milk, which increases turnover of ceramide to S1P by up-regulating ceramidase activity (162). In contrast to S1P, ceramide inhibits Akt signaling, which increases apoptosis and eNOS induction during vasculature remodeling in order to accommodate new growth prior to angiogenesis (161). This coordinated interplay of SM metabolites in the MFG establishes the architecture of blood vessels to meet the high metabolic demand of the expanding GI tract, aiding in gut maturation.
  Other factors involved in small intestinal development derived from the MFGM have been suggested. For example, lactoferrin (Lf) bi-directionally stimulates proliferation and differentiation of the small intestinal tissue by interacting with Lf receptors located on the enterocytes and crypt cells (163). Expression of the plasma membrane Lf receptor was shown to be highest in the small intestine (163).

### Intestinal immune maturation

When faced with an immune challenge, two key biological factors are involved in the intestinal defense response: the mucosal immune system and the gut microbiota (the latter will be discussed in the following section). A recent study reported that supplementation with bovine MFGM was able to enhance overall immunity and metabolism (10) which may explain the previously reported reduction in infection-related diarrhea (6). This section discusses potential mechanisms whereby components in the MFGM are able to regulate cellular events to enable maturation of the mucosal immune system, thereby improving infant health (Table 4).

**Table 4:** MFGM proteins and lipids involved in the infant intestinal immune system.

**MFGM protein**	**Functions**
α-lactalbumin	Proteolysis of α-lactalbumin generates peptides with bactericidal or immune-stimulatory activities (164, 165). Protects against diarrhea caused by enteropathogenic *Escherichia coli* (166).
Butyrophilin subfamily 1 member A1 (BTN1A1)	Involved in the regulation of lipid secretion (167). Involved in T-cell proliferation and metabolism (168).
Human leukocyte antigen II (HLAII)	May present maternal antigens to infant T-cells (169–171).
Lactadherin (PAS VI/VII, MFG-E8)	Regulates apoptosis by phagocytes (127, 172). Induces anti-inflammatory responses (173). Regulates T-cell proliferation and cytokine production profile by dendritic cells (173). Involved in the protective effect against rotavirus (119, 174).
Lysozyme	Inhibits the growth of Gram-negative bacteria by disrupting the outer membrane and cooperating with lactoferrin (175).
Mucin-1 (MUC1)	Binds to microorganisms and chemicals to prevent infection and inflammation (176). Inhibits the growth of *Salmonella enterica* serovar Typhimurium (177), S-fimbriated *Escherichia coli* (178), and rotavirus (179). Suppresses inflammation caused by *Pseudomonas aeruginosa* and its flagellin by down-regulating Toll-like receptor pathways (180, 181).
Osteopontin (OPN)	Binds to *Streptococcus agalactiae* and *Staphylococcus aureus*, enhancing phagocytosis by macrophages (162). Induces Th1 immune response (elevating IL-12 production from the macrophages) while suppressing the Th2 immune response (reducing IL-10 secretion) [162)].
Xanthine oxidoreductase (XOR)	Generates reactive oxygen species with antibacterial properties (182).
Gangliosides	Regulates activity and functionalities of immune cells including lymphocytes and dendritic cell, playing a role in developing immune tolerance (41).

While the immune system begins to develop *in utero*, there is rapid development after birth. Immediately following delivery, newborns have a limited capacity to initiate immune responses as the adaptive immune system is still influenced by the active suppression that occurs *in utero* to prevent adverse immunological reactions from occurring between the mother and fetus (183). In addition, this suppression enables the infant to develop tolerance against antigens such as breast milk proteins and commensal microorganisms after birth (184). For example, fetal CD4^+^ naïve T-cells tend to differentiate into Foxp3^+^ CD25^+^ regulatory T-cells (T_reg_) in fetal lymphoid tissue (185, 186), which suppresses antigen-specific immune reactions and inflammation. This active suppression of the adaptive immune system, combined with low exposure to antigens prior to birth (little immunological memory) (187, 188), and a sudden flow of food and microorganisms entering the gut after birth increases the risk of infections in newborns.

Maturation of adaptive immunity involves antigen exposure, followed by T-cell differentiation in response to antigens presented by antigen presenting cells (APCs). Human MFGM contains human leukocyte antigen II (HLAII) (97), an antigen presenting complex typically expressed on the surface of APCs that may be derived from HLA-DR (a subgroup of the major histocompatibility complex (MHC) Class II) on the mammary gland epithelium (169). Notably, only secretory epithelial cells in the lactating mammary gland, but not non-lactating cells, were found to express HLA-DR (169). Moreover, milk exosomal MHC Class II is more abundant during early lactation and gradually decreases, whereas MHC Class I shows the opposite trend (171). These studies suggest that HLAII on the MFGM may be involved in presenting antigens encountered by mothers to CD4^+^ T-cells in the infant gut, thereby supporting immune education during early life when tolerance is being established.

Some MFGM proteins may be involved in modulating properties of T-cells, the key regulator of the immune system. Butyrophilin (BTN) has been shown to negatively regulate T-cell proliferation and activity. When mouse CD4^+^ T-cells were activated by immobilized anti-CD3 antibody in the presence of the recombinant Fc fusion proteins BTN1A1 and BTN2A2, T-cell proliferation as well as IL-2 and IFN-γ production were inhibited (168). Thus, BTN in the mammary gland epithelium and the MFGM may control the function of maternal T-cells in the mammary gland and milk, respectively. They may also impact neonatal T-cells, although human BTN1A1 is digested rapidly in the infant stomach (102).

Lactadherin, also known as MFG-E8, is another MFGM protein with T-cell regulatory function. Lactadherin supplementation of formula led to the differentiation of naïve CD4^+^ T-cells to CD3^+^CD4^+^CD25^+^ T_reg_ in Peyer's patches of rat pup ileum, an important segment of the intestine that is involved in the immune response (173). In the same study, expression of OX62^+^CD4^+^SIRP^+^ dendritic cells (DCs) increased in Peyer's patches, which was coupled with an increase in production of the anti-inflammatory cytokine, IL-10 (173). These patterns in the T-cell population and cytokine production were shown to continue after weaning (173), suggesting a long-lasting effect of lactadherin supplementation. Previously, a positive correlation was observed between lactadherin concentrations in breast milk and protective effects against rotavirus infection (measured by morbidity) in Mexican infants, which was independent of the level of secretory IgA and other milk components such as BTN and mucin (119). This observation is supported by cell culture models showing that human lactadherin limits the infectivity of rotavirus in Caco-2 cells (174). Furthermore, lactadherin prevents tissue damage caused by prolonged inflammation by clearing apoptotic cells, thereby facilitating immune resolution (127, 172). To accomplish this, lactadherin binds to phosphatidylserine on the external membrane of apoptotic cells via its C-terminal V/VIII like domains, and its epidermal growth factor (EGF) domain contains the arginine-glycine-aspartate (RGD) motif that interacts with α_v_β_3_ and α_v_β_5_ integrin receptors of phagocytes (172). The binding of lactadherin to integrin receptors leads to activation of the signaling cascade that enables macrophages to engulf apoptotic cells (127). Delivery of lactadherin by the MFGM may be critical because active mitosis, which occurs during intestinal maturation, is accompanied by a high rate of apoptosis due to the many errors made in DNA replication and the subsequent clearance of mutated cells by the p53 protein (189).

Another minor MFGM protein associated with the developing immune system is milk alkaline phosphatase (AP), whose anti-inflammatory activity in the gut may contribute to protection against inflammation that is induced by the presence of large quantities of lipids from a high-fat diet (190). Although the role of AP in MFGM is not entirely clear, AP is endogenously produced by enterocytes, which, as a host defense mechanism, dephosphorylates pro-inflammatory molecules such as lipopolysaccharide (LPS), inhibiting TLR-mediated NFκB signaling and subsequent inflammation in the gut (190). Milk phospholipids and fatty acids, particularly LCFA, are strong stimulators of intestinal AP activity (190).

Osteopontin (OPN) is another minor MFGM protein that is involved in the development of both the innate and adaptive immune system of newborns. OPN functions as an opsonin, binding directly to bacteria such as *Streptococcus agalactiae* and *S. aureus* to enhance phagocytosis by macrophages (191). OPN is also involved in balancing the Th1 and Th2 immune responses as a cytokine, where it induces the Th1 immune response through elevation of IL-12 production from macrophages, while suppressing the Th2 immune response through lowering IL-10 secretion (192).

Milk lipids have been shown to interact with milk proteins during digestion, altering the types of peptides remaining in the gut, which in turn affect their bioactivities. In a piglet model, a formula incorporating both milk fat and vegetable oil stabilized by a protein-MFGM fragment mixture inhibited digestion of β-casein and β-lactoglobulin, thereby increasing the proximal jejunum and ileum content of immunoreactive peptides derived from those proteins. This was not observed in animals fed a formula incorporating vegetable oil with the same emulsifier nor a formula with vegetable oil stabilized only with a protein mixture (8, 9). This indicates that milk fat (mainly TAG) and differences in structural organization of molecules at the interface are the contributors for the observed modulatory effects.

Gangliosides in the MFGM are involved in multiple aspects of the mucosal immune system. Dietary gangliosides have been shown to modulate intestinal cytokine and IgA production (41) as well as lymphocyte activation (193). An inhibitory role of GM3 and GD3 on dendritic cell functionalities has also been reported (41), suggesting that in addition to promoting defense against aggressive antigens, milk gangliosides may promote tolerance against non-aggressive antigens, which is equally important during the first stages of life. GD3 levels in milk are higher in colostrum and GD3 has superior inhibitory activity against dendritic cell functions compared with GM3, indicating that immune modulation by gangliosides is more prominent during early infancy (41). This suggests that there may be a relationship between compositional changes in breast milk and infant gut maturation over the course of lactation.

### Limitations in studies of MFGM functions in infant immune development

There are some limitations in studies of MFGM and its roles in infant immune development. Most of the proposed immune mechanisms are based on studies focusing on isolated components of MFGM rather than intact MFGM. Indeed, the fate of MFGM through the neonatal and infant GI tract during digestion remains to be elucidated. In order to better understand how MFGM is able to modulate the intestine, future studies should examine how MFGM is digested, and in which part of the GI tract bioactive components of MFGM proteins are liberated from their complex structure. Furthermore, because the MFGM composition changes dynamically during lactation, and given that there is variability among individuals (102), identifying those components of the MFGM responsible for infant immune development would help move the field forward. Specifically, there is a lack of data concerning which components of MFGM in human milk are highly conserved and/or variable (not only in quality but also in quantity). This information may help us better understand the critical components of MFGM, and define which are important for maternal and/or infant health. For example, lactose exhibits very low variation (4% CV) among individuals, which suggests that this major osmotic component that regulates milk volume is highly conserved and may be important for the developing infant (194). Nonetheless, it is becoming more evident that MFGM proteins support the proper education and development of the immune system in infants.

## Impact on the infant gut microbiota

The human microbiota represents a community of commensal, symbiotic, and pathogenic microorganisms inhabiting the body (195). From the earliest moments in life, the microbiome is progressively built on a foundation initiated by key events, including delivery method (196), gestational age (197), antibiotic use (198), and nutrition (199), and eventually stabilizes into an adult-like state by approximately 12 months of age (198). The gut microbiota is dynamic during early life and is critically important for the maturing infant GI tract and the immune system as it establishes the basis for long-term metabolic and immune health (200). Nutrition remains a crucial factor during early development as dietary components are in constant interaction with the nascent microbiome, and emerging evidence suggests that the MFG, or components thereof, may contribute toward its evolution at this early stage of life. Structural differences between human MFG, which are enveloped by the MFGM, and infant formulas, which contain homogenized vegetable fats emulsified with dairy proteins and/or emulsifiers, influence the extent to which nutrients are digested in the intestine and have downstream effects on the gut microbiota. The following sections summarize evidence for a modulatory role of MFG and its components on the gut microbiota of the developing infant.

### MFG core lipids

The structure of human MFG and specific positional distribution of fatty acids may explain differences in the gut microbiota between breast-fed and formula-fed infants. Human milk contains β-16:0 (palmitic acid esterified on the *sn-2* position) in contrast to vegetable-sourced palmitic acid, which is esterified in the *sn*-1 or−3 positions (201). One study found that supplementing an infant formula with β-16:0 increased fecal abundance of *Lactobacillus* and *Bifidobacterium* genera in infants after 6 weeks of feeding compared to a control formula containing vegetable-sourced 16:0 (202). The mechanisms behind this observation need to be determined, but the position of palmitic acid on the TAG suggests that lipid structure may be important for gut microbiota. Similar bifidogenic results were shown with an infant formula supplemented with both β-16:0 and a mixture of prebiotic oligosaccharides (203) and formulas containing high amounts of β-16:0 with and without supplemented oligofructose (204).

In contrast to a formula containing only vegetable-derived lipids, a combination of milk fat and MFGM fragments altered fecal microbial composition in piglets by increasing *Proteobacteria* abundance at the expense of *Firmicutes* (which included the *Escherichia*/*Shigella*, and *Klebsiella* genera), as well as increasing *Bacteroidetes* (including members of the *Parabacteriodes* genus) (9). This may partly be explained by an increased intestinal content of immune modulatory peptides (as discussed earlier) as well as milk lipid-derived metabolites. However, studies on the gut microbial composition of breast-fed compared to formula-fed infants found the opposite effect, where a higher abundance of *Firmicutes* was observed in breast-fed infants, at the expense of *Proteobacteria* (205). Interestingly, differences have also been observed in the rate at which large and small MFG molecules are digested by pancreatic lipase, the former of which are hydrolyzed more slowly (206) likely affecting the accessibility of lipid products of the MFG core for bacterial metabolism.

Another study found that an infant formula high in MCFAs (coconut oil) emulsified with bovine MFGM (Lacprodan® MFGM-10) enriched the bacterial families *Bacteriodaceae, Desulfovibrionaceae, Rikenellaceae*, and *Porphyromonadaceae*, while formulas made with LCFAs emulsified with soy lecithin increased the abundance of *Enterobacteriaceae, Erysipelotrichaceae, Coriobacteriaceae*, and *Enterococcaceae* in the colon of germ-free mice (207). The effect of dietary fatty acids on the gut microbiota may also depend on the chain length and desaturation degree of the fatty acids (207). Human milk contains high amounts of medium chain fatty acids (207), such as C10:0 and C12:0, which have previously been shown to inhibit the growth of several strains of food-borne pathogens (208). Lipolysis of MFG core lipids is capable of causing cell lysis in undesirable microbes through the detergent-like characteristics of free fatty acids, and monoglycerides (113, 209).

An interesting feature of MFG lipids is that they can influence which protein digestion products enter the colon by altering the rate at which proteins are hydrolyzed in the small intestine (9) (as discussed earlier in section Intestinal Immune Maturation). A review on bacterial utilization of undigested luminal proteins and peptides was previously published (210). Overall, these results suggest that lipids derived from milk fat may indirectly enrich specific bacterial populations in the infant gut; however, additional studies are required to understand how this occurs.

### MFGM lipids

It is conceivable that remnants of the MFGM escaping digestion in the lumen proceed to the large intestine and support the colonization of microbial communities (15). Comparison of the colonic bacteria of germ-free mice fed two different types of emulsifiers, soy lecithin vs. MFGM phospholipids (Lacprodan® PL-20, Arla, Denmark), revealed that MFGM phospholipids tended to enrich *Porphyromonadaceae*, while soy lecithin tended to enrich *Enterobacteriaceae* and *Enterococcaceae*. Interestingly, mice fed MFGM phospholipids showed lower cecal concentrations of branched SCFAs (isovaleric acid, isobutyric acid), which are products of protein metabolism, compared to soy lecithin fed mice, suggesting a decrease in proteolytic activity in the ceca (207). In a separate study, rat pups fed a formula supplemented with bovine MFGM (Lacprodan® MFGM-10, Arla, Denmark) experienced an increased gut microbial species richness and evenness compared with rat pups fed a formula containing vegetable fat. At the phylum level, the microbiota of rat pups fed MFGM were more similar to the group reared on dam's milk, with similar levels of *Firmicutes* and *Proteobacteria*. In contrast, the control pups on regular formula experienced increased *Proteobacteria* and reduced *Firmicutes*. In the same study, *Lactobacilli* were determined to be the most abundant in dam-reared pups, present in pups supplemented with MFGM, but not detected in pups fed control formula (152).

In addition to supporting the growth of beneficial microbes, MFGM also exhibits antimicrobial activity that appears to protect against the development of infectious diseases. For example, a double-blind randomized controlled trial (RCT) showed that during the first 12 months of life, infants fed an experimental formula supplemented with bovine-derived MFGM (Lacprodan® MFGM-10) from 2 until 6 months of age experienced fewer acute otitis media (AOM) infections, and less fever than those receiving a control formula (7). These findings are supported by the observation that breast-fed infants experience lower rates of AOM in comparison to formula-fed infants (211, 212), and parallel another RCT in pre-school children (mean age 4.4 ± 0.9 y), which showed that consuming 200 mL chocolate milk containing an MFGM concentrate derived from bovine milk enriched with phospholipids (INPULSE®) daily for 4 months resulted in fewer days with fever, a reduction in fever incidence, and improved behavioral outcomes compared to children consuming a non-enriched chocolate milk control (213).

MFGM polar lipids such as sphingophospholipids and gangliosides have been shown to exhibit antimicrobial activities and protect against lipopolysaccharide-induced inflammation associated with Gram-negative bacteria (153, 214) and the development of colitis after *Clostridium difficile* infection (152). Degradation products of sphingomyelin, such as sphingosine, have shown bactericidal activities against specific bacteria (208, 215). Adult mice fed a high-fat diet supplemented with sphingomyelin sourced from bovine milk resulted in lower fecal Gram-negative bacteria while enriching bifidobacteria (216). In preterm newborns, infants fed a formula supplemented with bovine gangliosides resulted in reduced *E*. *coli* fecal counts and enriched *Bifidobacterium* compared to a standard formula (193).

### MFGM proteins

The protective mechanism behind the anti-infection activities associated with the MFG may also be related to MFGM proteins. For example, xanthine oxidase and antimicrobial proteins such as lactoferrin, lysozyme, and secretory immunoglobulin A (sIgA) are known antibacterial and/or antiviral proteins. Bovine MFGM has demonstrated antimicrobial activity, whose *in vitro* digestive products have been shown to selectively suppress the growth of *Salmonella typhimurium* (217).

Xanthine oxidoreductase (XOR), a major oxidative enzyme present in the MFGM, generates reactive oxidative species (ROS) such as hydrogen peroxide and superoxide anion, as well as reactive nitrogen species (RNS), which may play a role in the antimicrobial defense of the GI tract (122). A decoy effect of surface carbohydrates of XOR has also been suggested (122). Indeed, a growth inhibitory activity of XOR was demonstrated against *E*. *coli* (218) and *S*. *enteritides* (182). One interesting feature of XOR associated in immune defense of the infant is that infant saliva contains hypoxanthine and xanthine, substrates for XOR, in concentrations that are 10-fold higher than in adults, resulting in the generation of sufficient quantities of hydrogen peroxidase to inhibit the growth of *S*. *aureus* and *Salmonella* spp. (219). Interestingly, the enzyme activity of XOR in human milk peaks during the first few weeks of lactation likely as a protective measure for the immature gut at this stage, diminishing thereafter despite constant levels of protein expression (220). XOR is located in the inner leaflet of the MFGM, and its release and activation in the oral phase may provide a first line of defense against pathogen invasion. Indeed, a study on the oral microbiota in infants (<2 mo) given formula supplemented with bovine MFGM found that *Moraxella catarrhalis*, one of the most common bacteria associated with otitis, was less prevalent in oral swabs at 4 months of age compared to infants fed standard formula (221).

α-Lactalbumin, a minor protein embedded in the MFGM, is digested by pepsin, trypsin, and chymotrypsin during passage through the GI tract (97), generating bactericidal peptides (e.g., Gly-Leu-Phe; GLF peptides) (165) that protect from infection by enhancing macrophage phagocytosis and stimulating oxidative metabolism in neutrophils (164). Supplementation of infant formula with α-lactalbumin has previously shown protection against diarrhea by enteropathogenic *E. coli* in infant rhesus macaques (166). Another minor component of the MFGM, lysozyme, has potent bactericidal properties against both Gram-positive and -negative bacteria due to the presence of 1,4-β-N-acetylmuraminidase which can degrade bacterial cell walls (222). This action by lysozyme is supported by lactoferrin, which sequesters iron and directly interacts with the negatively charged Lipid A moiety of LPS to damage the bacterial membrane (114).

These data together provide evidence of the supportive effect of MFGM on the mucosal immune system, and that MFGM serves as a key component in milk enhancing intestinal defense during early life, while establishing stable commensals in the gut.

### MFGM glycobiome and the infant gut microbiota

The MFGM contains glycoconjugates (glycolipids and glycoproteins) harboring both *N*-linked and *O*-lined glycan moieties (12). About 70% of bovine glycolipids in milk are associated with the MFGM (223). The glycosylation patterns of these glycoconjugates in milk are tightly regulated by gene expression (98, 224), and determine resistance to digestive enzymes and functionality in the gut. The ability of certain bacteria to selectively bind to the intestinal mucosa through recognition of specific sugar moieties influences susceptibility to infection (11). The glycoproteins (MUC1, lactadherin) and gangliosides of the MFGM have the ability to interfere with pathogen recognition of, or attachment to, the intestinal mucosa, causing the pathogens to instead interface with the antimicrobial components embedded within the MFGM (225). For example, several glycoproteins derived from porcine MFGM were able to inhibit intestinal adhesion of *E. coli* F4ac (226). Mucin has been shown to inhibit the invasion of common enteric pathogenic bacteria such as the *Salmonella enterica serovar Typhimurium SL 1344* (177), S-fimbriated *Escherichia coli* (178), and rotavirus (179), which could partly explain the lower risk of *Salmonella* infection in breast-fed infants compared to formula-fed infants (177). The structural diversity of MFGM-bound oligosaccharides greatly differs between mammalian species (11, 146–148), and little is known about the functional differences. However, it is tempting to speculate that the differences have to do with the specificity of host-microbial interactions.

### Probiotic/prebiotic effects of MFGM in the infant gut

It has been hypothesized that probiotic bacteria (such as members of the *Lactobacillus* and *Bifidobacterium* genera) are able to pass from the mammary gland through to the infant colon by adhering to components of the MFGM, suggesting that the MFGM may be a probiotic carrier (227). Recently, Pannaraj et al. (228) established that bacteria found naturally in breast milk are able to seed the infant gut during the early stages of gut development (228). Another study showed that select OTUs assigned to *Bifidobacterium*, specifically *B*. *breve, B*. *bifidum*, and *B*. *longum*, were identified in breast milk and infant feces of the same mother-infant pair, but not in the oral cavity, suggesting that breast milk provides an important inoculum of specific bacteria for seeding the infant gut (229). Bacteria (including *Lactobacillus*) present in milk have been shown to preferentially associate with the MFGM utilizing glycan adhesion factors that enable them to bind to mucin (227). In addition, bacteria with greater surface hydrophobicity, such as *L*. *reuteri*, are better able to adhere to the MFGM (230), a phenomenon that is related to properties of the bacterial cell surface. In this context, several patents have been published for utilizing the MFGM as a probiotic carrier, which have recently been reviewed with a focus on lactic acid bacteria (227).

Mucin and lactadherin have been detected intact in gastric aspirate samples of pre-term infants fed breast milk, which suggests that MFGM glycoproteins remain stable and are able to survive gastric digestion (102). The MFGM may therefore confer a prebiotic effect, potentially providing a source of carbon to support the growth of the colonic bacteria (231). Indeed, members of both the *Ruminococcus* and *Bifidobacterium* genera are capable of producing extracellular glycosidases to digest glycans and glycolipids (232). Interestingly, *Lactobacillus paracasei*, and *Bifidobacterium* spp. isolated from cheese products were shown to survive in carbohydrate-restricted media by utilizing membrane-bound sugars on the MFGM as an energy source (233). This prebiotic effect is likely due to the sialic acid residues (234) on the gangliosides, which can be utilized by *B*. *infantis* and *B. bifidum* (231). This may account for the increased SCFAs observed during *in vitro* incubation of fecal material with MFGM isolates (235).

## Conclusions and future perspectives

MFGs are complex structures that are found in breast milk and growing evidence suggests a role for these important biomolecules in the early stages of human life. The observation that MFGs are heterogeneous in size and composition suggests that MFGs take on multiple roles in the developing neonate. It is known that the rate at which MFGs are digested is related to their diameter, and that proteins in the MFGM of some globules are able to resist pepsin hydrolysis better than others. Unique features of the MFGM, such as the lipid rafts, which are formed through the integration of cholesterol with highly saturated sphingolipids, create a rigid structure that enables minor components of the MFGM with bioactive properties to survive digestion. The extensive glycosylation of major and minor proteins found in the MFGM also appears to help them resist digestion, thereby enabling their passage to the colon intact. Thus, it is intriguing to consider that, in addition to their role as an energy-dense source of nutrition, the MFG may aid in the development of intestinal structure and the immune system, as well as the establishment of the intestinal microbiota in the neonate. Most studies published to date rely on data generated from animal models as the methods required to rigorously examine these research questions remain too invasive for human studies. However, important observations have already been made, including the finding that MFGM supplementation can accelerate intestinal development and improve intestinal integrity and vascular tone. Furthermore, several proteins associated with the MFGM are able to modulate the production and activity of immuno-modulatory components, such as T-cells, providing mechanistic evidence to support the role of MFG in development of the immune system. Finally, several animal studies and a growing number of studies involving infants show that the MFG and its components shift core microbial populations in the lower gut *via* multiple mechanisms, including the action of MFGM fragments that are resistant to digestion, through the unique distribution of fatty acids in the lipid core, and by antimicrobial activities associated with some MFGM components. Future studies should aim to elucidate the digestive fate of individual MFG and MFGM components to better understand their metabolic fate in different regions of the GI tract. As discussed, the MFG can be affected by maternal genetics, diet and environmental factors, and a deeper understanding of the connection between those factors, MFG composition, and downstream effects in the infant may improve dietary strategy of nursing mothers. Further, data on biological conservation as well as variations in MFG components observed among mammalian species (e.g., bovine vs. human) would provide guidelines for the development of infant formulas that meet the specific needs of human infants in cases where breastfeeding cannot be done. Overall, a growing body of literature continues to unravel the unique features of the MFG which suggest it plays important roles in preventing infection, supporting neurodevelopment, and shaping the maturing immune system and gut microbiota. These characteristics further underscore the importance of breast milk for the developing neonate.

## Author contributions

HL, EP, YH, JL, MP, and AW drafted the manuscript. OH, BL, and CS edited the manuscript.

### Conflict of interest statement

The authors declare that the research was conducted in the absence of any commercial or financial relationships that could be construed as a potential conflict of interest. The reviewer LM and handling Editor declared their shared affiliation.
